# Response of dissolved organic matter and bacterial community to anthropogenic disturbances in a plateau lake

**DOI:** 10.3389/fmicb.2025.1554202

**Published:** 2025-04-02

**Authors:** Yue Meng, Xin Jiang, Yue Li, Chun Qing, Xingyu Long, Pinhua Xia

**Affiliations:** ^1^Guizhou Province Key Laboratory for Information System of Mountainous Areas and Protection of Ecological Environment, Guizhou Normal University, Guiyang, China; ^2^Guizhou Key Laboratory of Plateau Wetland Conservation and Restoration, Guiyang, China; ^3^Guizhou Caohai National Nature Reserve Management Committee, Bijie, Guizhou, China; ^4^School of Chemistry and Materials Science, Guizhou Normal University, Guiyang, China

**Keywords:** anthropogenic disturbances, assembly process, dissolved organic matter, microbiome, lake

## Abstract

**Introduction:**

Dissolved organic matter (DOM) and bacterial communities play essential roles in lake ecosystem biogeochemical cycles. However, the effects of anthropogenic disturbances on their interactions are not fully understood.

**Methods:**

This study used UV-vis techniques, excitation-emission matrix parallel factor analysis, and 16S rRNA sequencing to reveal the differences in the structures of fluorescent DOM (FDOM) and bacterial communities in lake sediments and water under different levels of anthropogenic disturbances. Methods such as Spearman correlation analysis, null model, neutral community model and random forest analysis were explored how FDOM composition and bacterial communities respond to anthropogenic disturbances in the sediments and water of the Caohai Lake.

**Results:**

The results indicated that sediment FDOM was sensitive to anthropogenic disturbances, with protein-like substances dominating heavily disturbed areas (69%) and humic-like substances dominating less disturbed areas (63%). However, no significant difference in FDOM composition was found in the water. Similarly, α and β diversity indices for bacterial communities showed no marked variation (*P* > 0.05) between highly and lightly disturbed areas in both water and sediment samples. Nevertheless, co-occurrence network analysis revealed more negatively correlated links and longer average path length with stronger disturbances. This suggests that while the intensity of anthropogenic disturbance has not yet reached a threshold sufficient to alter the structure of the bacterial community, it might have influenced the types and quantities of resources accessible to the community. Consequently, bacteria might have responded to these changes through competitive interactions, enabling them to resist environmental fluctuations. We found that anthropogenic disturbances were positively linked stochastic processes in the bacterial community assembly and influenced groups that degraded terrestrial humic-like substances. Moreover, the sources and fluorescence components of DOM could have shaped bacterial diversity and community assembly.

**Discussion:**

Overall, these findings illustrate that anthropogenic disturbance affects FDOM composition and its relationship with bacteria, providing valuable insights for managing shallow lake ecosystems.

## 1 Introduction

In recent decades, intensified human activities have introduced large volumes of industrial, agricultural, and domestic wastewater into rivers, lakes, coastal areas, and other aquatic ecosystems, profoundly altering their biogeochemical cycles and leading to severe ecological consequences (He et al., 2011). For instance, nutrient loading in urban lakes reduces oxygen levels and biodiversity, often causing algal blooms (Funkey et al., 2014). Dissolved organic matter (DOM) is the most active component of organic materials and is a key energy source in aquatic systems. When microorganisms degrade highly bioavailable DOM, dissolved oxygen is significantly depleted, which can lead to anoxic conditions and indirectly affect lake ecosystems. DOM also directly affects lake ecosystems by potentially disrupting plant photosynthesis and causing physiological harm, which can lead to sediment conditions that are unfavorable for plant growth and bacterial community development (Reitsema et al., 2018). Additionally, DOM’s complex array of functional groups and chemical bonds of DOM influences pollutant transport, transformation, and bioavailability in lake ecosystems through mechanisms such as absorption and complexation (Bai et al., 2017; Ding et al., 2022; Wen et al., 2024).

Research has shown that anthropogenic disturbances are the primary drivers influencing the amount, source, and composition of DOM in lakes (He et al., 2022; Massicotte et al., 2017; Roebuck et al., 2020). For example, Ifon et al. (2023) suggested that urban land use that replaced the natural grasslands and forests in the Jiulong River basin increased protein-like substances in DOM and enhanced its bioavailability. A comparative study of DOM in the Laurentian Great Lakes revealed that rural and vegetated lakes had higher DOM concentrations and diversity, while urban and open waters had lower levels (Larson et al., 2014). Additionally, Zhou et al. (2022) examined DOM composition and anthropogenic impacts on river water quality across four major Chinese rivers and observed that human activities significantly influenced the molecular composition and bioavailability of DOM.

Bacteria are essential for lake ecosystem decomposition processes, facilitating carbon cycling and supporting the balance of aquatic ecosystems (Ren et al., 2013). Human-induced changes such as land use, urbanization, industrialization, and agriculture can affect the spatial distribution, co-occurrence networks, and assembly processes of bacterial communities (Wu et al., 2022). Industrial wastewater discharge inhibits the growth of photosynthetic microorganisms in lakes and promotes the growth of anaerobic saprophytic bacteria (Grossart et al., 2019). Nutrients from aquaculture increase the nutrient load in water bodies, leading to the homogenization of fungal and bacterial community structures (Geng et al., 2022). Additionally, agricultural wastewater can fragment bacterial communities, thereby reducing the ecosystem services provided (Kraemer et al., 2020).

Dissolved organic matter and bacteria are integral to lake ecosystems with a complex, interdependent relationship. Microbial metabolites are species-specific, meaning that changes in bacterial composition can influence DOM’s chemical diversity of DOM. Conversely, the bacterial community assembly is affected by the chemical composition of DOM, as different bacteria utilize different types of DOM (Li et al., 2023). DOM also mediates between anthropogenic disturbances and bacterial dynamics. In the downstream areas severely affected by human interference, the input of pollutants alters the composition of natural DOM, influencing the microbial degradation process and nutrient cycling (Wang et al., 2024). For instance, Zhang et al. (2020) discovered that in the water of urban rivers impacted by human activities, protein-like components are the primary constituents of the DOM structure, with the structure and composition of DOM being crucial factors influencing the structure of the bacterial community. Overall, the DOM-bacteria relationship is intricate. It plays a crucial role in water quality and the health of freshwater ecosystems, making it an important area of current research. It’s necessary to further explore the impact of anthropogenic disturbances on this relationship, particularly within single shallow lake systems.

Caohai Lake is located in Guizhou Province, China and is a typical karst plateau wetland ecosystem. It is also a shallow lake, with high loadings of organic matter content found in the lake sediments which is sensitive to environmental changes and anthropogenic disturbances (Dai et al., 2017; Xia et al., 2020). The county town of Weining lies near the northeastern side of the lake, and until 2017, approximately 8,000 tons of municipal wastewater were discharged into Caohai Lake daily (Long et al., 2021). Local tourism relies heavily on Caohai Lake’s Xihai and Jiangjiawan Marinas, both positioned on the northeastern side, whereas the southwest side of the lake, distant from the city, experiences lower population density and reduced anthropogenic impacts, including agriculture and animal husbandry (Chen et al., 2016; Hu et al., 2020). Based on varying levels of human activity, the northeastern side of the Caohai Lake is classified as a high-disturbance area, whereas the southwestern side is considered a low-disturbance area. This distinction makes the Caohai Lake an ideal natural study area for examining the effects of anthropogenic disturbances on lake DOM composition and bacterial communities. In our study, DOM composition and bacterial community structure in lake sediments and overlying water across these disturbance zones were analyzed using three-dimensional excitation emission fluorescence spectroscopy and Parallel factor analysis PARAFAC (EEM-PARAFAC) and 16S rRNA high-throughput sequencing. This study aimed to (1) assess how anthropogenic disturbances could affect fluorescent DOM (FDOM) and bacterial diversity in lakes and (2) explore how these disturbances could influence the relationship between FDOM and bacterial communities.

## 2 Materials and methods

### 2.1 Sampling site

Caohai Lake (26°49′N–26°53′N, 104°12′E–104°18′E), is situated in southern Weining County, Guizhou Province, China, in the hinterlands of the Wumeng Mountain Area of the Yunnan–Guizhou Plateau at an altitude 2171.7 m (Zhang et al., 2022). The lake comprises a typical plateau wetland ecosystem and covers an area of about 25 km^2^, with an average temperature of 10.5°C and average of annual sunshine hours at 1805.4 h. It has a subtropical plateau monsoon climate (Liu J. et al., 2024). Several rivers, including the Dazhong, Wanxia, Baima, and Dongshan Rivers, flow into the lake, and along the way, they receive various domestic, industrial, and agricultural wastewaters. Among them, the Dazhong River has a relatively high level of pollution, and human activities in its basin are also the most intense (Xia et al., 2016; Yang et al., 2021).

**FIGURE 1:**
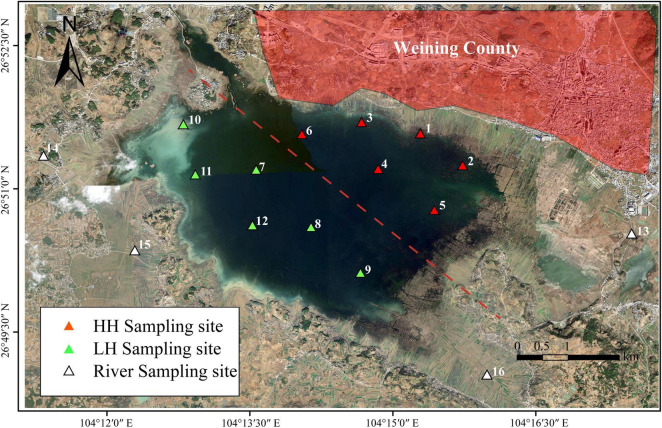
Distribution of sampling points. “HH” represents the high anthropogenic disturbed area, “LH” represents the low anthropogenic disturbed area, and “R” represents the river area.

Points 1–12 were within the lake area (Figure 1), with points 1–6 located in the northeastern region near Weining and the marinas, which is a densely populated area with relatively frequent anthropogenic activities. It is reported that the construction of sewage treatment plants and pipeline networks in this area has long lagged behind, and the capacity for harmless treatment of domestic waste is weak, leading to the direct discharge of domestic sewage and landfill leachate into the Caohai Lake. Meanwhile, tourism activities in this area, supported by the Xihai Marina, Jiangjiawan Marina, and Baijiazui Marina, are intense (Long, 2022). Despite the area being closed to the public, a large amount of tourist-generated domestic waste remains, causing significant disturbance to the ecological environment of the Caohai Lake. Points 7–12 were in the southwestern region, influenced by secondary anthropogenic activities, such as agriculture and cattle and sheep stockbreeding activities (Wang et al., 2019; Xu and Gao, 2009), and were classified as the LH area. Points 13–16 were situated in the Dazhong, Wanxia, Dongshan, and Baima Rivers, respectively. The samples were collected during the summer (August, 2023) because this season represents a critical period for hydrological and ecological dynamics in the study area. At first, due to the plateau monsoon climate of Caohai, the higher summer temperatures (average of 23.6°C) enhance microbial activity and on the other hand, due to the impact of increasing human activity linked to tourism, making it an ideal period to study the variations in microorganisms and DOM. The water samples were collected at a depth of 0.5 m, with 3 L taken from each point. Surface sediments (0–10 cm) were collected at similar water depth (WD) using a mechanical grabbing instrument, with three sediment samples from each point combined and stored in sterile sealed bags at 4°C.

### 2.2 Sample measurements

An HQ4300 portable water quality analyzer was used to measure water temperature (WT), pH, oxidation-reduction potential (ORP), electrical conductivity (EC), and dissolved oxygen (DO). Sediments ORP, and pH were measured using a soil redox potential instrument (HENGMEI, HM-YH). The total nitrogen (WTN), total phosphorus (WTP), Chlorophyll a (Chl.a), and permanganate index (COD_*Mn*_) in the overlying water were analyzed following to the standard methods (Jin and Tu, 1990). Sediments samples were freeze-dried and sieved through a 100-mesh sieve. Total nitrogen (STN) was determined by the Total Kjeldahl Nitrogen (STKN) method while total phosphorus (STP) was analyzed using the digestion-Mo-Sb spectrophotometric approach (Bao, 2000).

### 2.3 DNA extraction and high-throughput amplicon sequencing

Following the manufacturer’s instructions, Water and sediment DNA were extracted using the E.Z.N.A.^®^ soil DNA Kit (Omega Bio-tek, Norcross, GA, United States). The concentration and purity of the extracted DNA were assessed using a NanoDropTM 2000 spectrophotometer (Thermo Fisher Scientific, United States) and analyzed on 1% agarose gels. To identify bacteria, the hypervariable region V3–V4 of the 16S rRNA gene was amplified using universal primers 341F/806R, following previous protocols (Ran et al., 2023). High-throughput sequencing was conducted at Tianjin Nuohe Zhiyuan Bioinformation Technology Co., LTD, Tianjin Province, China. The original DNA sequences were quality-controlled using the QIIME2 (V2020.2) program. Redundant sequences were removed, and the remaining data were denoised to generate representative sequences of amplicon sequence variants (ASVs) and the corresponding ASVs abundance table.

### 2.4 DOM fluorescence spectroscopy, UV-vis spectroscopy and DOC analysis

Organic carbon analysis required filtration of 20 mL of surface water through a GF/F filters (0.45 μm pore size, 47 mm diameter, combusted for 4 h at 450°C). A total of 4 g of sediment powder was put into a centrifuge tube, and then 40 mL of ultrapure water was added. The mixture was shaken at 220 rpm for 16 h at 20°C and then centrifuged for 15 min at 4,500 rpm. The supernatant was filtered using a glass filter to obtain sediment DOM filtrates (Zhao et al., 2022). Dissolved organic carbon concentrations (DOC) were measured using a TOC analyzer (TOC-L-CPH, SHIMADZU, Japan). The excitation-emission matrices (EEMs) of filtrates were analyzed using Horiba Aqualog^®^ fluorescence spectrometer, employing a 1 cm quartz cuvette. The parameter settings for spectral scanning are shown in Table 1.

**TABLE 1:** 3D-excitation-emission matrix (EEM) spectral scanning parameter settings.

Parameter type	Parameter setting
PMT voltage	700 V
Lamp	150 W Xenon arc lamp
Steps	Ex = 2 nm, Em = 2 pixels
Excitation and Emission slit width	2 nm
Scanning signal integration time	2 s

All EEMs were blank subtracted (Milli Q water) to correct Raman and Rayleigh scatter effects, and corrected for inner-filter effect following the Aqualog Operational Manual. The Raman scattering emission peak was obtained by measuring Milli-Q water, which was used to normalize the fluorescence intensity to Raman units (R.U.) (Du et al., 2022). A UV-Visible spectrophotometer was also used for scanning, with ultrapure water used as the blank and by scanning with a 10 mm Quartz Cell in the range of 230–800 nm, at an interval of 1 nm (Li et al., 2015).

### 2.5 Data processing and analysis

The Parallel Factor analysis (PARAFAC) was performed in MATLAB R2024a using the “drEEM” toolbox (Murphy et al., 2013). Parallel factor analysis according to Pitta and Zeri (2021). At first, the spectral data were preprocessed. Since no significant DOM fluorescence signals were observed at emission wavelengths above 600 nm, the emission range between 599.428 and 827.571 nm was excluded prior to analysis. Additionally, noticeable noise was observed in some excitation-emission matrices (EEMs), so the excitation wavelengths below 250 nm (240–245 nm) were also removed from all datasets. Furthermore, the smoothing function in the “drEEM” toolbox was utilized to interpolate the first- and second-order Rayleigh and Raman scattering bands. This function adapts to the signal intensity and the extent of residual scattering across different datasets. Water and sediment samples were analyzed together in PARAFAC, and in order to obtain accurate information on fluorescence composition, PARAFAC modeling was carried out from 4 to 8 components, where the four components can be validated according to split-half analyses, residual analysis and loadings. Finally, the data was compared with the OpenFluor online spectrum database^1^ to infer the composition of obtained components. The fluorescence spectra were then applied to compute the fluorescence index (FI) (McKnight et al., 2001), humification index (HIX) (Lee et al., 2019), and biological index (BIX) (Huguet et al., 2009), which describe DOM composition (Gao et al., 2022). The UV absorbance at 254 nm dividing by the DOC concentration (SUVA_254_) was positively related to DOM aromatic content (Weishaar et al., 2003). SUVA_254_ > 4 indicates a predominantly hydrophobic, whereas the SUVA_254_ < 3 suggests a primarily hydrophilic component (Edzwald and Tobiason, 1999). The spectral slope ratio (SR) defined as the ratio of the UV absorbance at 275–295 nm to that at 350–400 nm, is inversely proportional to the molecular weight of DOM (Liu et al., 2018) and is often used to describe its molecular weight and source (He et al., 2016). Statistical analysis was performed using Excel and SPSS26, while R studio and Origin Pro2023 were utilized for data visualizations. The α-diversity of bacterial communities was calculated using the “vegan” package. The non-metric multidimensional scaling (NMDS) was utilized to analyze the spatial pattern of microbial communities. To explore the process of community assembly, the mean nearest taxon distance (βMNTD) and the β nearest taxon index (βNTI) were calculated based on the (Xun et al., 2019) method. A Neutral Community Model (NCM) was constructed following the method described by Sloan et al. (2006). Correlation and random forest analysis were conducted using the “ggplot2” and “random forest” packages, respectively.

## 3 Results

### 3.1 Environmental parameters of sediments and water

The environmental factors of the water and sediment in Caohai Lake are presented in Supplementary Tables 1, 2, respectively. No significant differences were observed in the overlying water for the various parameters except for temperature and N/P ratio. The N/P ratio in the HH area was significantly lower than in the LH area. The concentration of Chl.a in HH area was higher than that in LH area, indicating a greater algal biomass in the former. Although there was no statistically significant difference, the average TP content in the HH area was twice that of the LH area. The Chl.a and COD_Mn_ content in rivers are significantly lower than those in lake areas, but the TP content is significantly higher than that in lake areas. The results of the One-way ANOVA indicate that there were no significant differences in the physicochemical properties of the sediments in the lake area. However, the ORP and pH of the river sediments were significantly higher than those of the lake sediments, while the TN was significantly lower than that of the lake sediments. The DOC concentration in the overlying water of Caohai Lake ranged from 10.45 to 14.23 mg/L, with the average of 13.07 mg/L which was comparable to the DOC concentration in the entering rivers (13.27 mg/L). In contrast, the DOC concentration in lake sediments ranged from 40.05 to 154.02 mg/L, with the average of 86.62 mg/L, which was higher than that in river sediments (51.33 mg/L).

### 3.2 FDOM composition, spatial distribution and source analysis in sediment and water

**FIGURE 2:**
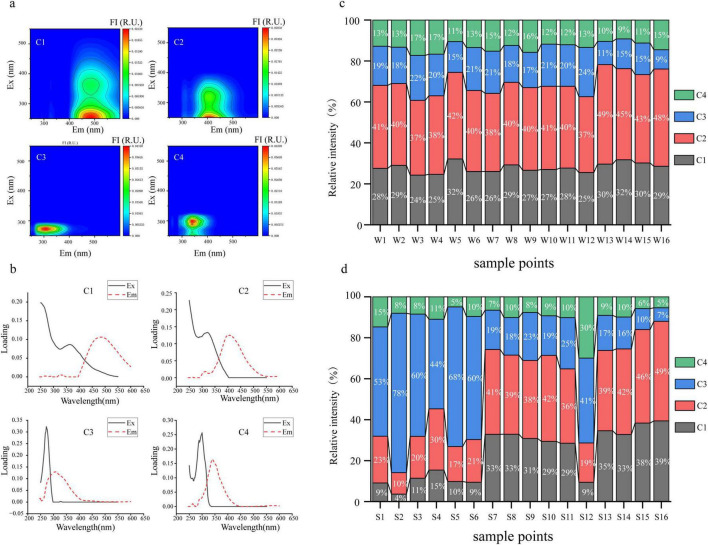
Characteristics of the DOM. **(a)** Excitation-emission matrix (EEM) contour plots of the four fluorescent components (C1 to C4) identified by PARAFAC; **(b)** Loadings of the four fluorescent components (C1 to C4) identified by PARAFAC; **(c)** Percentage of DOM components in the overlying waters; **(d)** Percentage of DOM components in the sediments. (C1, terrestrial humic-like component; C2, microbial humic-like component; C3, tyrosine-like component; C4, tryptophan-like component).

**FIGURE 3:**
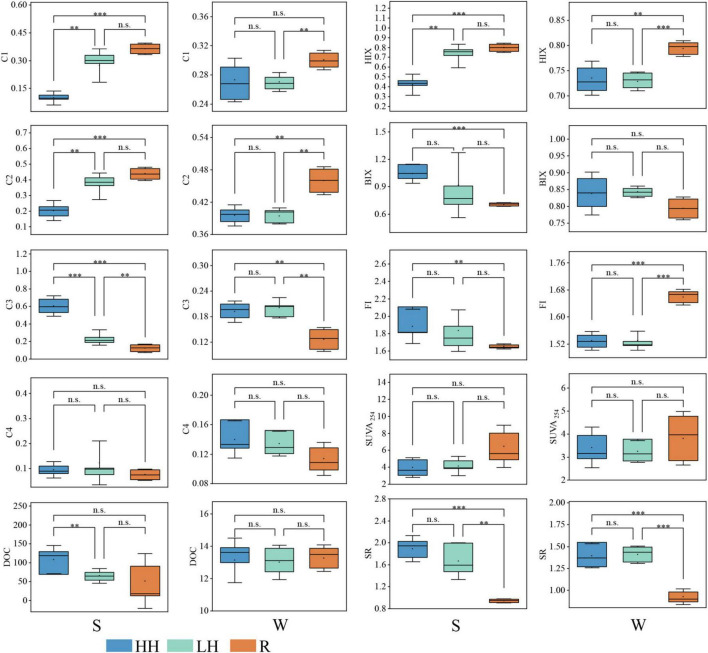
Box plots and T-test of the fluorescent DOM components (C1: terrestrial humic-like component; C2: microbial humic-like component; C3: tyrosine-like component; C4: tryptophan-like component), fluorescence index (FI), biotic index (BIX), humic index (HIX), aromatization index (SUVA_254_), and slope rate (SR) of water and sediment. DOC, dissolved organic carbon. “HH” represents the high anthropogenic disturbed area, “LH” represents the low anthropogenic disturbed area, and “R” represents the river area, “S” represents sediment sampling, “W” represents water sampling. (** indicates *p* < 0.005, *** indicates *p* < 0.001, ns indicates *p* > 0.05).

The PARAFAC model (Figures 2a, b and Supplementary Table 3) identified four components in the sediment and water: C1 (terrestrial humic-like), C2 (microbial humic-like), C3 (tyrosine-like) and C4 (tryptophan-like). The results indicated that the FDOM in both sediment and water primarily consisted of these components. The spatial distribution of DOM fluorescence components in Caohai Lake sediments exhibited notable differences (Figures 2d, 3), with the C1 and C2 contents being significantly higher in the LH area than HH area. The FDOM in river sediments has a similar composition to that in the LH area, and the content of C3 is significantly lower than that in the HH area and the LH area, with humic-like DOM being the main component.

Conversely, relative C3 content was significantly higher in the HH region than in the LH region. In the overlying water (Figures 2c, 3), humic-like substances dominated, accounting for approximately 67% of the total FDOM, while in riverine input, humic-like components accounted for approximately 76%. The contents of C1 and C2 are significantly higher than those in the LH area, and the content of C2 is significantly higher than that in the HH area. The content of C3 is significantly lower than that in the HH area and the LH area (Figure 3). This indicates that the main component input into the lake through the river is humic-like substances. Although the DOC content in the overlying water exhibited minimal spatial variation, the sediment DOC content demonstrated a pronounced range with higher levels in the HH area than in the LH area (Figure 3). The SUVA_254_ values of the lake surface waters ranged from 2.4 to 4.9 (Figure 3), with an average of 3.3, whereas the lake sediments had values ranging from 2.5 to 5.9, with an average of 4.1. These findings suggest that FDOM in the Caohai Lake is primarily composed of hydrophobic substances (Edzwald and Tobiason, 1,999).

The SR of waters and sediments in rivers entering Caohai Lake were significantly lower than in the lake. This indicates that the DOM in the rivers contains a large amount of components with high molecular weight, strong aromaticity, and originating from vascular plants (He et al., 2016). After entering the lake, it may be transformed into low molecular weight organic matter through microbial or light mediated processes (Ning et al., 2021; Song et al., 2019). The fluorescence parameters could offer further insight into the source, humic content and freshness of FDOM. A higher BIX suggests a greater input of biological or aquatic bacterial organic matter, whereas a lower BIX indicates a greater input of terrestrial matter (Zhang et al., 2023). The FI is commonly employed to identify the source of FDOM, with the values less than 1.4 suggesting that terrestrial sources as the primary origin and values exceeding 1.9 indicating that endogenous FDOM predominates (Gao et al., 2022). The results in Figure 3 revealed that FDOM in the overlying water and sediment was both terrestrial and endogenous, with the majority being exogenous. The HIX, which represents the degree of FDOM humification, was 0.75 in water and 0.57 in sediment, with all sampling points below four, which demonstrated a relatively low level of humification in the FDOM. In the overlying water, there were no significant differences in FI, BIX, HIX, SR, and SUVA_254_ between the HH area and the LH area. The HIX and FI of the rivers flowing into the lake were significantly higher than those of the lake area, with the average value of FI being 1.66, indicating that humic-like substances were the dominant components entering the lake area from the rivers. The HIX of the sediments in the HH area was significantly lower than that of the LH area and the rivers flowing into the lake, while the BIX and FI were significantly higher than those of the rivers flowing into the lake. These results are consistent with those in Figure 2d, suggesting that the sediments in the HH area contain a higher content of endogenous organic matter such as protein-like substances.

### 3.3 Bacterial community structure

High-throughput 16S rRNA sequencing technology was used to obtain 147,655 valid DNA sequences, representing over 98% of the bacterial community. This depth of sequencing ensured that the DNA database met the required standards and provided sufficient data for further analyses. A total of 65 phyla, 163 classes, 363 orders, 507 families, 1,051 genera, and 734 species of microorganisms were identified in the water and sediment of the Caohai Lake.

**FIGURE 4:**
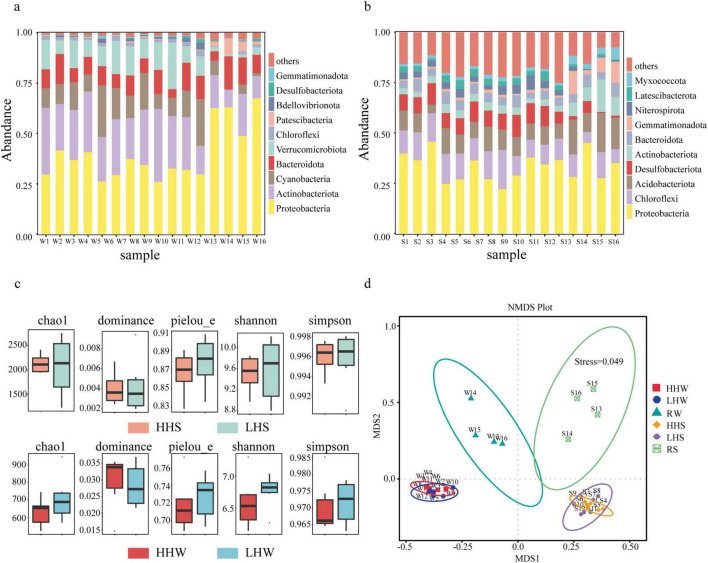
Relative abundances of bacterial taxa in waters of Caohi Lake. **(a)** Histogram showing the relative abundances of bacterial taxa in sediments. **(b)** Alpha diversity index values of communities. **(c)** NMDS analysis of bacterial community composition. **(d)** HHS: sediments from anthropogenically active areas; LHS: sediments from less anthropogenically active areas; HHW: waters from anthropogenically active areas; LHW: waters from less anthropogenically active areas.

The average Chao value for the microbial communities in the water (Figure 4a) of the lake area and the four entering rivers was 944.654. The Shannon and Simpson indices averaged 7.14 and 0.98, respectively, while the Pielou evenness index averaged 0.74. At the phylum level (Figure 4a), the bacterial structures of the surface waters in the lake were similar, with dominant phyla including *Proteobacteria* (26%–42%), *Actinobacteria* (14%–36%), *Cyanobacteria* (7%–25%), and *Verrucomicrobiota* (7%–23%). The dominant classes were *Gammaproteobacteria* (7%–17%), *Actinobacteria* (13%–32%), *Alphaproteobacteria* (16%–31%), *Cyanobacteria* (7%–25%), *Verrucomicrobia* (7%–23%), and *Bacteroidia* (4%–15%). Among the four rivers entering the lake, *Proteobacteria* (49%–67%) and *Actinobacteria* (9%–21%) were the most dominant phyla.

The average Chao value for bacterial communities in the sediments (Figure 4b) of Caohai lake and its entering rivers was 2166.15, with average Shannon and Simpson index values of 9.68 and 0.996, respectively. The mean Pielou evenness was 0.88. In the lake sediments, the dominant phyla were *Proteobacteria* (22%–42%), *Chloroflexi* (8%–20%), *Acidobacteriota* (4%–12%), and *Desulfobacterota* (6%–11%) (Figure 4b). Bacterial community structures varied across different sampling points. In the sediments of the entering rivers, the dominant phyla were *Proteobacteria* (27%–45%), *Chloroflexi* (5%–13%), *Acidobacteriota* (9%–19%), and *Actinobacteriota* (7%–16%).

The NMDS analysis (Figure 4d) indicated significant differences in the bacterial communities of the sediment and surface water between the four rivers and the lake. However, no significant differences in bacterial α-diversity indices (Figure 4c) were observed between the HH and LH areas, suggesting a weak relationship between community diversity and human disturbance.

**FIGURE 5:**
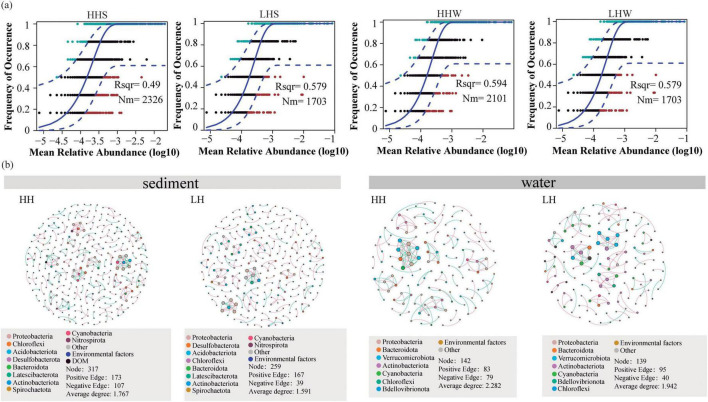
Fit of the neutral community model based on bacteria in two areas **(a)**. The overall goodness of fit of neutral community model was quantified using the “R^2^” value, where a higher R^2^ indicates a greater impact of randomness on community structure. The “Nm” parameter was used to assess the degree of diffusion among communities. Different colors represent ASVs that occur more or less frequently than predicted by the neutral community model. The blue solid line represents the model’s best fit. The blue dashed line represents the model’s 95% confidence interval. The Co-occurrence network interactions of bacteria communities, DOM composition, and Environmental factors in the sediment and surface water of the HH and LH areas **(b)**. “HH” represents the high anthropogenic disturbed area, and “LH” represents the low anthropogenic disturbed area.

The neutral model results (Figure 5) indicated that the R^2^ and Nm values of sediments from HH area are 0.49 and 2,326, respectively, while those from LH area are 0.361 and 1,495. Additionally, the water in HH area (Nm = 2101) exhibited a higher degree of bacterial species diffusion than the LH area (Nm = 1703). These results indicating that the anthropogenic disturbances led to a wider distribution of bacterial community in sediments, with a distribution pattern closer to that of the neutral model.

A co-occurrence network analysis was conducted to examine the impact of anthropogenic disturbances on the relationships between bacterial communities, environmental factors, and DOM. The results revealed regional differences with 317, 259, 142, and 139 nodes (nodes is the bacterial ASVs) in sediments and overlying water in the HH and LH areas, respectively. Positive correlations dominated the co-occurrence network. The sediment bacterial co-occurrence network in the HH area exhibited a higher average path length than the LH area, suggesting that bacteria in areas with anthropogenic disturbances were more sensitive to environmental fluctuations. Furthermore, the higher average degree of the nodes in HH area indicated that the relationships among bacteria and between bacteria and environmental factors were more complex in aquatic environments with high human disturbance (Zhai et al., 2024).

### 3.4 Relationship between DOM and bacterial community

**FIGURE 6:**
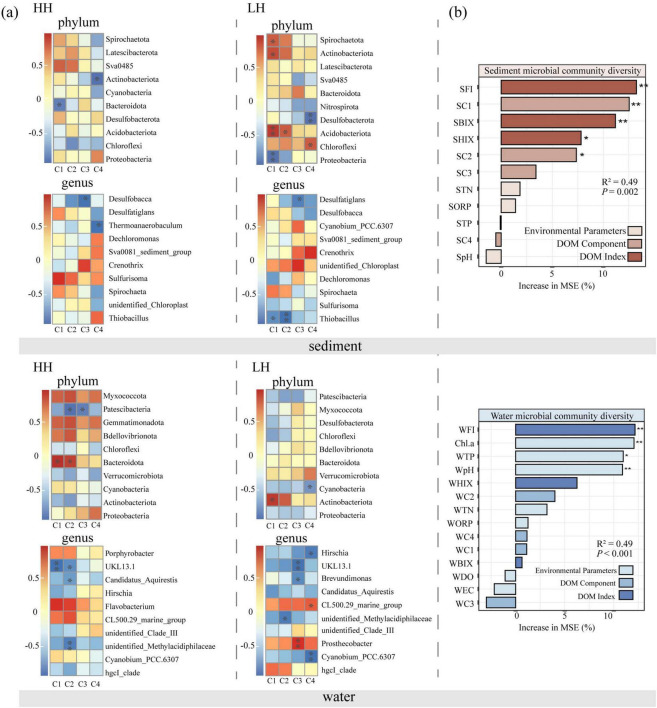
Correlations between fluorescent DOM components and fluorescent indices and bacterial abundance at phylum/genus level, with statistical significance indicated by asterisks **(a)**. “HH” represents the high anthropogenic disturbed area, and “LH” represents the low anthropogenic disturbed area. The importance of environmental parameters, DOM components and indices to sediment and water microbial community diversity in random forest analysis **(b)**. (* indicates *p* < 0.05; ** indicates *p* < 0.01).

Spearman correlation analysis was performed to examine the relationship between bacteria and DOM fluorescent components at the phylum/genus level within the study site (Figure 6a). At the phylum level, the results indicated that FDOM composition and structure were significantly associated with *Actinobacteria*, *Patescibacteria*, *Bacteroidota*, *Desulfobacterota*, and *Chloroflexi* in the overlying waters. In the sediments, *Proteobacteria*, *Actinobacteriota*, *Bacteroidota*, *Desulfobacterota*, and *Spirochaetota* had significant correlation to FDOM structure, and bacteria linked to the structural changes in FDOM varied between the two areas. In the sediments, C1 was negatively correlated with *Bacteroidota* in the HH area and with *Proteobacteria* in LH area, respectively; In the water of HH area, C1, C2, and *Bacteroidota* were positively correlated. However, this positive correlation was not found in the LH area. At genus level, *Thiobacillus* is significantly negatively correlated with C1 and C2 fluorescent components in LH area sediment, but no such trend was observed in HH sediment. In HH area, C3 fluorescent components is significantly negatively correlated with *Desulfobacca*, while in the LH area is significantly negatively correlated with *Desulfatiglans*. *UKL13-1* is significantly negatively correlated with C1 and C2 fluorescent components in the overlying water of HH area, but significantly negatively correlated with C3 fluorescent component in LH area. Although the correlation between hgcI-clade and C1, C2 fluorescent component in two areas is not significant, it shows an opposite correlation. Random forest analysis explained 49% of the variance in bacterial diversity in both sediment and water (Figure 6b). In sediment, FI, BIX, HIX and fluorescent component (C1 and C2) were the dominant drivers for bacterial diversity (*P* = 0.002), while in water, environmental parameters (Chl.a, TP, and pH) and FI were the vital predictors (*P* < 0.001).

To further investigate the relationship between bacterial community assembly and FDOM composition, we calculated | βNTI| values using the null model, evaluated the distances between FDOM components using Euclidean distances, and examined the relationship between | βNTI| values and FDOM components (Table 2). The results indicated that stochastic influences accounted for only 29% of the variation, whereas deterministic factors accounted for 71%, suggesting that deterministic selection predominates in the assembly of bacterial communities in sediments. In contrast, 79% of the variation in the phylogenetic composition of microbial communities in water was attributed to random processes. A strong negative correlation (*P* < 0.001) revealed that the C1 and C2 components were the primary determinants linked to bacterial community formation in sediment. Additionally, the C1 component was identified as a significant determinant of the bacterial community assembly in surface water, based on a strong negative correlation (*P* < 0.001).

**TABLE 2:** Spearman correlation analysis between the |βNTI| of sediment and overlying water taxa and the Euclidean distance of different fluorescent dissolved organic matter (FDOM) components in sediment and water.

FDOM Components	Sediment		Water	
	*P*-value	r	*P*-value	r
C1	−0.233	0.000	−0.217	0.013
C2	−0.221	0.000	−0.151	0.114
C3	−0.014	0.488	0.093	0.537
C4	−0.320	0.005	−0.052	0.934

C1, terrestrial humic-like component; C2, microbial humic-like component; C3, tyrosine-like component; C4, tryptophan-like component.

## 4 Discussion

### 4.1 Influence of anthropogenic disturbances on DOM composition and structure

Anthropogenic disturbances (such as domestic sewage, industrial sewage, agricultural runoff, etc.) can directly change the composition of FDOM through direct discharge (Yang et al., 2015), or it can indirectly change the FDOM content by increasing the nutrient load of the water (Cohen et al., 2014; Murphy et al., 2011), which stimulates the growth of phytoplankton and autotrophic bacteria, thereby producing FDOM (Du et al., 2021). The composition and structure of FDOM in sediments from the two areas showed significant differences. The C3 component accounted for 78% in the HH area compared to only 19% in the LH area. Domestic sewage contains high levels of protein and bio-unstable FDOM (Baker, 2001; Lan et al., 2022). The drainage outlet of the municipal sewage treatment plant in Weining County is located in the east of Caohai Lake. After treatment, the municipal domestic wastewater is discharged into Caohai Lake. FDOM, especially tyrosine-like substances, are not completely removed by conventional wastewater treatment processes (Du et al., 2021; Goldman et al., 2012). Furthermore, the local economic level is underdeveloped, and the sewage treatment facilities are still imperfect (Peng et al., 2020). As a result, there are some untreated domestic sewages still discharged into Caohai Lake. Therefore, the high content of tyrosine-like substances in the HH area may be attributed to the direct discharge from human activities.

Tyrosine-like substances were not only found in domestic sewage but also the algal degradation (Fellman et al., 2011). Bao et al. (2023) reported that the content of tyrosine-like substances in sediments increased during the blue-green algae bloom season in Chao Lake, suggesting that tyrosine-like substances was related to the release of algae-derived FDOM (Drake et al., 2015). In the HH area closer to the wharf and city, anthropogenic disturbances were more pronounced, and the BIX index in sediments was significantly higher than that in the LH area, indicating more microbial transformation and organic matter decomposition. Consequently, a large portion of FDOM in this area could be recent and primarily of biological origin. It can be concluded that the high load of domestic sewage stimulates bacteria or planktonic algae to produce biological origin FDOM, which is also one of the reasons for the high content of tyrosine-like substances in the HH area. Similar patterns have been observed for several lake types across China (Gao et al., 2024; Zhang et al., 2010).

FDOM at sampling point 12 was mainly composed of C3 and C4, accounting 71% (Figure 2d), and the content of C4 is 30%. Research shows that the wastewater from stockbreeding and agriculture contains a large amount of protein-like components, such as tryptophan-like and tyrosine-like components (Baker, 2002). Therefore, it can be speculated that this sampling point is affected by the non-point source pollution from agriculture and stockbreeding (Hudson et al., 2007). Since it is not located at the water inlet, the dilution effect is weak, and the exchange of nutrients is slow. As a result, more tryptophan-like and tyrosine-like components have accumulated (Wu Y. et al., 2024). In sediment, Tryptophan-like and tyrosine-like fluorescence are frequently attributed to the biodegradation processes of algae and bacteria (Hu et al., 2019). At the same time, the BIX, SR and the sediment TP content at this point are higher than those of other sampling points in this area, which means that the nutrient level here is high, promoting the activity of microorganisms and plankton, and causing a further accumulation of tryptophan-like components.

The composition of FDOM In the overlying water and sediment Is closely linked to the environmental conditions in the study area, with anthropogenic disturbance identified as a key factor influencing FDOM composition. Technologies such as FT-ICR-MS can be used to improve the precision of DOM detection at a molecular level. Future studies should focus on employing FT-ICR-MS to identify DOM sources and provide a clearer understanding of how anthropogenic disturbances drive changes in DOM within lake ecosystems.

### 4.2 Effects of anthropogenic disturbance on bacterial community

Our study found no significant differences in bacterial diversity between the two areas, suggesting that the level of disturbance around the Caohai Lake has not yet significantly affected bacterial communities. According to Wang et al. (2020), the microbial communities in Baiyang Lake remained similar under minor and moderate anthropogenic disturbances but changed under high disturbance. Caohai Lake is a shallow lake with an area of only 25 km^2^ and a water depth ranging from 0.3 m to 1.2 m and is highly susceptible to wind and wave disturbances, exhibiting a high degree of material exchange. Therefore, the absence of significant differences in bacterial communities between the two areas may be attributed to the high level of substance exchange in the lake.

Further analysis indicated that anthropogenic disturbances increased stochastic processes in the bacterial community assembly in the sediment. These stochastic processes were further amplified by urban sewage, industrial discharge, and agricultural activities but did not surpass the deterministic effect. Deterministic processes can drive communities to select more efficient species for resources utilization, as previously reported, whereas stochastic processes may increase the proportion of non-functional groups (Zhang et al., 2019). In contrast, the environmental conditions in the LH area were more stable and less influenced by anthropogenic disturbances, fostering a more consistent environment that supported the proliferation of specific bacterial groups and led to more predictable and structured bacterial communities.

Network analyses allow for the assessment of the relationship between bacterial communities and key indicators (Berry and Widder, 2014). In this study, no statistically significant differences were observed in the bacterial community composition between the two areas. However, the bacterial co-occurrence network in the HH area was more complex, particularly regarding in the number of negatively correlated edges and increase in the average degree. The LH area, with its more abundant vegetation and relatively stable ecosystem, contrasted with the HH area, which experienced higher levels of anthropogenic disturbances and pollutants. A positive correlation suggests a high degree of niche overlapping or positive interactions between taxa, whereas a negative correlation indicates distinct niches and negative interactions (Kurtz et al., 2015). Strong positive correlations between species were reported to enhance taxon synchronicity (Hernandez et al., 2021). An ecological community with many synchronous groups may be unstable. To illustrate, in a community with numerous strong and constructive connections, these members may respond in unison to environmental changes, thereby creating a resonant effect. The extinction of a key member puts the remaining members at risk as they are positively associated with that member. An increase in negative correlations between species can promote ecosystem stability because the reduced synchronization among species fosters compensatory dynamics, where a decrease in one species leads to an increase in another (Coyte et al., 2015). Anthropogenic disturbances may increase the selection pressure on microbial communities, enhance the interactions between bacterial communities (higher connectivity, more nodes, and connections), reduce the number of co-occurrence network modules, and merge them into fewer and larger modules (Wu M. et al., 2024). This may facilitate bacterial cooperation in response to environmental disturbances (Romdhane et al., 2022).

The regulatory role of climatic factors in shaping bacterial communities warrants scientific attention. For example, the climate in Caohai Lake, Guizhou belongs to the subtropical plateau monsoon climate. The rainfall from May to October accounts for approximately 88% of the annual precipitation (Yang et al., 2021). Therefore, the surface runoff caused by rainfall can bring organic matter and bacteria from the ground into lakes, which may affect bacterial communities (Le et al., 2016).

### 4.3 Effects of anthropogenic disturbances on the connection between bacterial community and DOM

The composition, structure and molecular weight of DOM are the key factors that influence its bioavailability and reactivity. Generally, amino acids, low-molecular-weight sugars, dissolved organic nitrogen and phosphorus, and DOM with a high O-C ratio are relatively stable, whereas humic substances and low molecular weight fatty compounds are bio-refractory (Lønborg et al., 2009). *Proteobacteria* and *Bacteroidota* are often found to be major bacterial OM degraders (Chen et al., 2023). They are the predominant bacteria in both areas, exhibiting a wide range of niches and the capacity to rapidly multiply and form dominance under various environmental conditions. At the phylum level, the C1 component was negatively correlated with *Proteobacteria* in the LH sediment but negatively correlated with *Bacteroidota* in the HH sediment (Figure 6). These findings suggest that in environments with less anthropogenic disturbance, *Proteobacteria* play a dominant role in the decomposition of terrestrial humic-like matter, contributing significantly to the formation of DOM structures. Conversely, in environment with strong anthropogenic disturbance, *Bacteroidota* dominated the degradation of terrestrial humic-like substances.

At genus level, we observed that the bacteria negative correlated with C3 were different in the sediments of the two area, in HH and LH area, they are *Desulfobacta* and *Desulfatiglans*, respectively. *Desulfobaca* has been found to be associated with the degradation of polycyclic aromatic hydrocarbons (PAHs) (Yan et al., 2015) and is believed to promote anaerobic methane oxidation in paddy soil, thereby reducing methane emissions (Ji et al., 2020). *Desulfatiglans* is a sulfate reducing bacterium (Liu Q. et al., 2024) that can obtain energy by coupling the oxidation of organic compounds and the reduction of sulfates (Qiu et al., 2019). An increase in the abundance of this bacterium promotes the degradation of sulfonamides. Interestingly, in the overlying water, we observed that *hgcI-clade* exhibited opposite correlations with humic-like substances (C1 and C2) in two areas.

Previous research has suggested that anthropogenic disturbances can significantly influence the dynamics of DOM and bacterial communities, particularly through the introduction of a wide range of organic and inorganic pollutants, which enhances the metabolic activity of microorganisms involved in the degradation and transformation of complex organic compounds (Wang et al., 2024). The relationship between DOM and bacteria is bidirectional and complex. The Microbial metabolism products are species-specific, so assembly of microbial communities drives DOM production and consumption. Li et al. (2023) found that DOM chemical diversity did not alter bacterial community assembly, but the latter shaped the former and altered the geographical distribution of DOM chemical diversity. Conversely, other researchers have argued that the DOM composition can affect microbial community diversity (Jimenez-Sanchez et al., 2015; Muscarella et al., 2019; Zhou et al., 2024). Their conclusion was based on the premise that the specific composition of DOM shapes particular niches, thereby favoring the assembly of specific bacterial communities. Random forests analysis revealed that the source of FDOM significantly affected the diversity of bacterial communities in both sediment and water. Additionally, the | βNTI| value of the bacterial community exhibited a negative correlation with the Euclidean distance of terrestrial humic-like substances, indicating that the stochastic process of bacterial community assembly becomes more prominent as the content of terrestrial humic-like substances increases (Zhou et al., 2021).

Compared with physical and chemical properties, anthropogenic disturbances have a more pronounced impact on the sediment FDOM in Caohai Lake. In both sediments and water bodies, the FI is the primary factor driving changes in the diversity index of the bacterial community (Figure 6). Therefore, we believe that anthropogenic disturbances can further influence the diversity and assembly process of the bacterial community by altering the quantity and composition of FDOM.

## 5 Conclusion

This study demonstrated how anthropogenic disturbances affected the composition and structure of FDOM and bacterial communities in lakes. Anthropogenic disturbances increased the input of protein-like substances into the lake, stimulating the bacterial and planktonic activities that transformed or produced protein-like matter and which was deposited in the sediment. However, owing to the high material exchange in shallow lakes, FDOM in the water remained relatively uniform. The introduction of organic and inorganic pollutants intensified the selection pressure, leading to greater competition or cooperation among bacterial communities to cope with environmental fluctuations. Furthermore, the bacterial groups responsible for degrading terrestrial humic-like substances varied with disturbance intensity, and the source and composition of FDOM were the key factors that influenced bacterial community diversity and structure. This suggested that FDOM mediated the relationship between anthropogenic disturbances and bacterial communities. Human-induced interference altered the composition and structure of FDOM, which affected the interaction between FDOM and bacteria. Given the complexity of this relationship, future studies should further explore the impact of human interference on the relationship between FDOM and bacteria. More accurate detection methods, such as FT-ICR-MS, stable isotope analysis, and biomarkers, can be considered to characterize the DOM at the molecular level, using metagenomics, qPCR, etc., to analyze the bacterial response from multiple angles.

## Data Availability

The 16S rRNA sequencing datas for this study can be found in the NCBI (Accession Number: PRJNA1204461).

## Footnotes

1
https://openfluor.lablicate.com
