# Supplementary material for: Response of dissolved organic matter and bacterial community to anthropogenic disturbances in a plateau lake
Source: Front Microbiol. 2025 Apr 2;16:1554202. doi: 10.3389/fmicb.2025.1554202 (PMC12000888; doi:10.3389/fmicb.2025.1554202)
Supplement: Supplementary file 1 [file Data_Sheet_1.docx]

***Supplementary Materials***

**Supplementary materials: 3 pages, 3 Tables.**

**List of Supplemental files**

Supplementary information (Table S1-S3) accompanies this paper.

Table captions

**Tab S1.** Physical and chemical properties of water.

**Tab S2**. Physical and chemical properties of sediment.

**Tab S3**. Characteristics of four PARAFAC components identified in DOM of Caohai Lake.

| Table S1 Physical and chemical properties of water （mean ±SD） | | | |
| --- | --- | --- | --- |
| Environmental parameters | HH | LH | R |
| WT（℃） | 22.35±0.61^a^ | 24.11±1.05^b^ | 20.62±0.26^c^ |
| WD (cm) | 116.5±22.85^a^ | 111.67±13.96^a^ | 118.25±12.03^a^ |
| DO （mg/L） | 7.74± 0.17^a^ | 7.51±0.49^a^ | 7.62±0.57^a^ |
| ORP (mV) | 401.05 ±35.30^a^ | 370.73 ±58.57^a^ | 371.70 ±22.53^a^ |
| EC (μs/cm) | 433.17 ±22.11^a^ | 440.33 ±39.10^a^ | 439.50±50.76^a^ |
| pH | 7.90 ±0.73^a^ | 8.24 ± 0.22^a^ | 8.19 ±0.11^a^ |
| WTN (mg/L) | 1.21±0.26^a^ | 1.32±0.20^a^ | 1.45±0.23^a^ |
| WTP (mg/L) | 0.04±0.01^a^ | 0.02±0.00^a^ | 0.17±0.07^b^ |
| N/P ratio | 31.50±6.16^a^ | 50.96±4.50^b^ | 10.58±2.77^c^ |
| COD_Mn_ (mg/L) | 6.80 ± 0.88^a^ | 6.77± 0.87^a^ | 3.98±0.77^b^ |
| Chl.a (mg/m³) | 14.70 ±2.67^a^ | 12.66± 2.67^a^ | 4.88±2.31^b^ |
| DOC (mg/L) | 13.13 ±1.26^a^ | 13.01 ±0.97^a^ | 13.27± 0.71^a^ |
| Different letters in the same line indicate a statistically significant difference（ANOVA, *P*<0.05）  “HH” represents the high anthropogenic disturbed area, “LH” represents the low anthropogenic disturbed area and “R” represents the rivers. | | | |

| Table S2 Physical and chemical properties of sediment （mean ±SD） | | | |  |
| --- | --- | --- | --- | --- |
| Environmental parameters | HH | LH | R | |
| ORP (mV) | -118.10 ±30.27^a^ | -167.00±62.99^a^ | 9.8 ±89.91^b^ | |
| pH | 6.74 ±0.13^a^ | 6.72 ±0.12^a^ | 7.71 ±0.16^b^ | |
| DOC (mg/L) | 107.88±34.05^a^ | 47.67±17.71^a^ | 51.33±58.49^a^ | |
| STKN （mg/g） | 11.01 ± 2.84^a^ | 9.53 ±3.63^a^ | 1.72 ±0.09^b^ | |
| STP （mg/g） | 0.47±0.08^a^ | 0.49 ± 0.10^a^ | 0.43 ±0.10^a^ | |
| Different letters in the same line indicate a statistically significant difference（ANOVA, *P*<0.05）  “HH” represents the high anthropogenic disturbed area, “LH” represents the low anthropogenic disturbed area and “R” represents the rivers. | | | |  |

| Table S3 Characteristics of four PARAFAC components identified in DOM of Caohai Lake. | | | |
| --- | --- | --- | --- |
| Component | Excitation / Emission maxima | | Description |
| C1 | 250-360/485 nm | Terrestrial humic-like fluorescence, Peak ‘A+C’ (Kulkarni et al., 2019; Li et al., 2016; Yan et al., 2020; Zhuang et al., 2022) | |
| C2 | 250-320/406 nm | Biological/Microbial humic-like fluorescence, Peak ‘M’ (Cawley et al., 2012; Chen et al., 2018; Garcia et al., 2018) | |
| C3 | 272/309 nm | Tyrosine-like fluorescence, Peak ‘B’ (Catalá et al., 2015; Dainard et al., 2015; Yamashita et al., 2013) | |
| C4 | 300/340 nm | Tryptophan-like fluorescence, Peak ‘T’ (Coulson et al., 2022; Murphy et al., 2011; Stedmon et al., 2011; Yamashita et al., 2013) | |

**References**

Catalá, T.S., Reche, I., Fuentes-Lema, A., Romera-Castillo, C., Nieto-Cid, M., Ortega-Retuerta, E., et al., 2015. Turnover time of fluorescent dissolved organic matter in the dark global ocean. Nature Communications. 6. 5986. <https://doi.org/10.1038/ncomms6986>.

Cawley, K.M., Ding, Y., Fourqurean, J., Jaffé, R., 2012. Characterising the sources and fate of dissolved organic matter in Shark Bay, Australia: a preliminary study using optical properties and stable carbon isotopes %J Marine and Freshwater Research. 63. 1098-1107. <https://doi.org/10.1071/MF12028>.

Chen, M., Jung, J., Lee, Y.K., Hur, J., 2018. Surface accumulation of low molecular weight dissolved organic matter in surface waters and horizontal off-shelf spreading of nutrients and humic-like fluorescence in the Chukchi Sea of the Arctic Ocean. Science of The Total Environment. 639. 624-632. <https://doi.org/10.1016/j.scitotenv.2018.05.205>.

Coulson, L.E., Weigelhofer, G., Gill, S., Hein, T., Griebler, C., Schelker, J., 2022. Small rain events during drought alter sediment dissolved organic carbon leaching and respiration in intermittent stream sediments. Biogeochemistry. 159. 159-178. <https://doi.org/10.1007/s10533-022-00919-7>.

Dainard, P.G., Guéguen, C., McDonald, N., Williams, W.J., 2015. Photobleaching of fluorescent dissolved organic matter in Beaufort Sea and North Atlantic Subtropical Gyre. Marine Chemistry. 177. 630-637. <https://doi.org/10.1016/j.marchem.2015.10.004>.

Garcia, R.D., Diéguez, M.d.C., Gerea, M., Garcia, P.E., Reissig, M., 2018. Characterisation and reactivity continuum of dissolved organic matter in forested headwater catchments of Andean Patagonia. Freshwater Biology. 63. 1049-1062. <https://doi.org/10.1111/fwb.13114>.

Kulkarni, H., Mladenov, N., Datta, S., 2019. Effects of acidification on the optical properties of dissolved organic matter from high and low arsenic groundwater and surface water. Science of The Total Environment. 653. 1326-1332. <https://doi.org/10.1016/j.scitotenv.2018.11.040>.

Li, P., Lee, S.H., Lee, S.H., Lee, J.-B., Lee, Y.K., Shin, H.-S., et al., 2016. Seasonal and storm-driven changes in chemical composition of dissolved organic matter: a case study of a reservoir and its forested tributaries. Environmental Science and Pollution Research. 23. 24834-24845. <https://doi.org/10.1007/s11356-016-7720-z>.

Murphy, K.R., Hambly, A., Singh, S., Henderson, R.K., Baker, A., Stuetz, R., et al., 2011. Organic Matter Fluorescence in Municipal Water Recycling Schemes: Toward a Unified PARAFAC Model. Environmental Science & Technology. 45. 2909-2916. <https://doi.org/10.1021/es103015e>.

Stedmon, C.A., Thomas, D.N., Papadimitriou, S., Granskog, M.A., Dieckmann, G.S., 2011. Using fluorescence to characterize dissolved organic matter in Antarctic sea ice brines. Journal of Geophysical Research: Biogeosciences. 116. <https://doi.org/10.1029/2011JG001716>.

Yamashita, Y., Boyer, J.N., Jaffé, R., 2013. Evaluating the distribution of terrestrial dissolved organic matter in a complex coastal ecosystem using fluorescence spectroscopy. Continental Shelf Research. 66. 136-144. <https://doi.org/10.1016/j.csr.2013.06.010>.

Yan, C., Sheng, Y., Ju, M., Ding, C., Li, Q., Luo, Z., et al., 2020. Relationship between the characterization of natural colloids and metal elements in surface waters. Environmental Science and Pollution Research. 27. 31872-31883. <https://doi.org/10.1007/s11356-020-09500-x>.

Zhuang, W.-E., Chen, W., Yang, L., 2022. Effects of Photodegradation on the Optical Indices of Chromophoric Dissolved Organic Matter from Typical Sources. International Journal of Environmental Research and Public Health. 19. 14268. <https://doi.org/10.3390/ijerph192114268>.
